# Adverse Pregnancy Outcomes with Co-Occurring Opioid and Stimulant Use Disorders

**DOI:** 10.3390/jcm15082811

**Published:** 2026-04-08

**Authors:** Alexandra R. Schroeder, Noor Al-Hammadi, Tucker Doiron, Niraj R. Chavan

**Affiliations:** 1 Division of Maternal Fetal Medicine, Department of Obstetrics, Gynecology, and Women’s Health, SSM Health Saint Louis University, St. Louis, MO 63117, USA; 2 Department of Health and Clinical Outcomes Research, Advanced HEAlth Data (AHEAD) Research Institute, Saint Louis University, St. Louis, MO 63103, USA

**Keywords:** substance use, opioid, stimulant, APO, adverse pregnancy outcome

## Abstract

**Background/Objectives**: Substance use disorder (SUD) in pregnancy is an increasingly complex public health challenge that is known to worsen maternal and neonatal outcomes. Rates of polysubstance use are steadily rising. The objective of this study was to assess the impact of co-occurring opioid and stimulant use disorder on adverse pregnancy outcomes (APOs) among inpatient pregnancy hospitalizations. **Methods**: We conducted a cross-sectional analysis of inpatient pregnancy hospitalizations for delivery admissions from the Healthcare Cost and Utilization Project (HCUP) National Inpatient Sample (NIS) from 2016 to 2020. ICD-10 codes were used to identify patients with opioid and stimulant use disorder and with APOs. APO was defined as a composite to include hypertensive disorders of pregnancy, antepartum hemorrhage, postpartum hemorrhage, preterm birth, and fetal growth restriction. Multivariable logistic regression analyses were undertaken to predict the likelihood of APOs among pregnancy hospitalizations with opioid use, stimulant use, or co-occurring (opioid and stimulant) use disorders. Sociodemographic covariates, including age, race and/or ethnicity, insurance payor type, and income level, were accounted for. **Results**: From 2016 to 2020, 32,602 delivery hospitalizations complicated by stimulant or opioid use disorder were identified. Of these admissions, 21,049 (64.6%) had opioid use disorder, 9472 (29.1%) had stimulant use disorder, and 2081 (6.4%) had co-occurring opioid and stimulant use disorder. In the entire cohort, the prevalence of APOs was significantly highest among pregnancy delivery hospitalizations with co-occurring opioid use and stimulant use disorder (1136/2081—54.6%, *p* < 0.001), as compared with opioid use disorder (8923/21,049—42.4%) or stimulant use disorder alone (4654/9472—49.1%). Rates of APOs increased in subsequent years for all cohort groups. Adjusting for relevant sociodemographic covariates, co-occurring opioid and stimulant use disorder was an independent predictor of APO (aOR 3.65; CI 95%, 3.34–3.99). In comparison, opioid use disorder and stimulant use disorder were independent predictors of APOs with a less strong correlation, aOR 2.22 (CI 95%, 2.16–2.29) and aOR 2.89 (CI 95%, 2.77–3.02), respectively. **Conclusions**: Patients with co-occurring opioid and stimulant use disorder have the highest exposure risk for APOs, acting as an independent predictor for APOs when adjusting for sociodemographic covariates.

## 1. Introduction

Substance use disorder (SUD) in pregnancy is an increasingly complex public health challenge that is known to worsen maternal and neonatal outcomes. The Diagnostic and Statistical Manual of Mental Disorders, Fifth Edition, defines SUD as a “cluster of cognitive, behavioral, and physiological symptoms indicating that the individual continues using the substance despite significant substance-related problems [1].” In patients with SUD, pregnancy is a particularly challenging time with increased exposure to health care, pressure for cessation, and heightened risks for both mother and baby [2,3,4,5]. In addition, pregnant patients with SUD have a higher risk of severe maternal morbidity (SMM) [4,6,7,8,9,10,11], in addition to increased risk for adverse neonatal outcomes, including heightened rate of congenital anomalies, preterm birth, low birth weight, and long-term adverse neurological effects [6,12,13,14,15,16,17].

Opioids are medications and/or drugs that are used medically in the treatment of pain. These medications are used outside of clinical settings through illegal use to produce symptoms of euphoria and decreased pain perception. Opioid drug use includes a wide range of substances, including prescription narcotic medications (e.g., oxycodone, hydrocodone, and morphine) and illegal opioids (e.g., heroin and illicitly manufactured fentanyl). Opioid drugs act on various receptors throughout the nervous system to block nociception and produce an analgesic effect [18]. Opioid use during pregnancy is associated with maternal lack of prenatal care, placental abruption, overdose, respiratory depression, and death. Fetal risks to maternal opioid use include risks of neonatal withdrawal symptoms, neonatal intensive care unit admission, fetal growth restriction, preterm labor, and fetal death, with mixed evidence on increased congenital anomalies [19].

Stimulants are a category of medications and/or drugs that increase the activity of the central nervous system. They are used in the clinical setting for treatment of mental health disorders such as attention deficit hyperactivity disorder or narcolepsy, among other uses. They are additionally used recreationally, legally or illegally, for performance enhancement and euphoria. Stimulant drugs include caffeine, amphetamines, methylphenidate, ephedrine, pseudoephedrine, and modafinil. Stimulant drugs act by increasing catecholamine levels and targeting adrenergic receptors [20]. Stimulant use in pregnancy is associated with maternal cardiovascular complications, including hypertension and myocardial ischemia, increased likelihood for intensive care admission, and need for a cesarean section. Fetal risks to maternal stimulant use include fetal death, possible increased risk of congenital anomalies, preterm delivery, fetal growth restriction/low birth weight, and potential for withdrawal symptoms [7].

Previous cross-sectional research evaluating the relationship between various types of SUDs and adverse pregnancy outcomes (APOs) has noted opioid and stimulant use as the most commonly identified substances during pregnancy hospitalizations when excluding tobacco and cannabis use [11]. However, current patterns of substance use point toward the increasing widespread prevalence of co-occurring substance use disorders (i.e., polysubstance use) during pregnancy, of which some of the highest increases are noted with stimulant and tobacco use [21].

As the opioid epidemic has escalated over the last decade, research interest in the perinatal impact of SUD has also expanded [16,19,22,23]. In contrast to the abundant research on the impact of opioid use in pregnancy [7,24,25,26], the impact of stimulant use and the potential impact of co-occurring opioid and stimulant use disorders remain understudied. Given the increasing prevalence of stimulant use in general as well as stimulant use in the perinatal context [27], elucidating the differential impact of opioid and stimulant use versus co-occurring stimulant and opioid use on adverse perinatal outcomes is critical for risk stratification and patient counseling. Hence, this study was undertaken to evaluate the impact of co-occurring opioid and stimulant use disorder on APOs among inpatient pregnancy hospitalizations using the Healthcare Cost and Utilization Project (HCUP) National Inpatient Sample (NIS).

## 2. Materials and Methods

### 2.1. Study Design

We performed a cross-sectional analysis of the HCUP-NIS, the largest publicly available all-payor database released yearly in the United States (Agency for Healthcare Research and Quality, Rockville, MD, USA). The HCUP-NIS provides national estimates of inpatient utilization, access, cost, quality, and outcomes with diagnosis and procedure codes, with a weighted evaluation of 35 million hospitalizations annually [28]. This study used the 2016 through 2020 NIS datasets. The study years were selected to follow the transition from ICD-9 to ICD-10 coding standards, thereby avoiding inaccuracies associated with code conversion. The NIS database is de-identified, and this study was deemed exempt by our Institutional Review Board.

### 2.2. Study Population

Hospital admissions for pregnant persons were identified using ICD-10 diagnosis and procedure codes pertaining to an ongoing pregnancy. Diagnosis-Related Group (DRG) codes were additionally used to specifically identify admissions associated with a delivery episode, in order to avoid duplication of patients with multiple inpatient hospitalizations over the course of the same pregnancy. Delivery hospitalizations were subsequently categorized into three mutually exclusive exposure cohorts: (1) opioid use disorder only, (2) stimulant use disorder only, and (3) co-occurring opioid and stimulant use disorder. ICD-10 codes used to identify opioid use disorder, stimulant use disorder, and adverse pregnancy outcomes are provided in Appendix A. Stimulant use disorder ICD-10 codes included both codes for cocaine use and for other stimulant use.

### 2.3. Study Criteria

Inclusion criteria: Inpatient delivery hospitalizations from 2016 to 2020 with a valid DRG delivery code and a diagnosis of opioid use disorder, stimulant use disorder, or co-occurring opioid and stimulant use disorder based on ICD-10 coding.

Exclusion criteria: Hospitalizations without a delivery-associated DRG code, hospitalizations with no identified SUD or with a SUD other than opioid or stimulant use disorder, and records with missing data in any variable of interest were excluded from the analysis.

**Figure 1:**
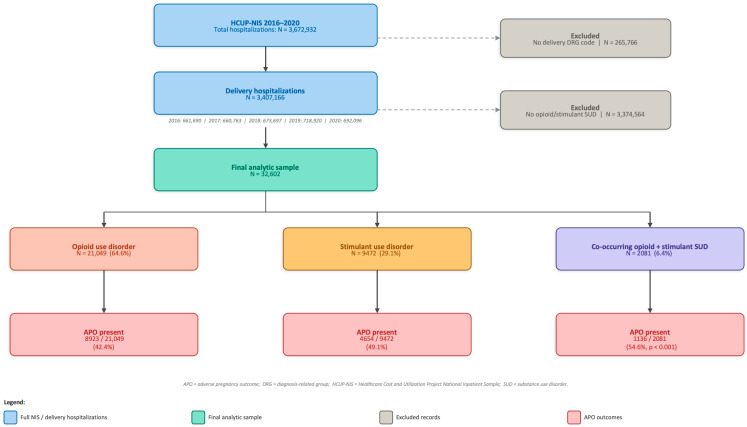
Study Selection Flowchart.

The study selection process is illustrated in Figure 1. Briefly, the full NIS across 2016–2020 comprised 3,672,932 hospitalizations. After excluding admissions without a delivery DRG code (*n* = 265,766), a total of 3,407,166 delivery hospitalizations were identified. Following further exclusion of hospitalizations without opioid or stimulant use disorder (*n* = 3,374,564), the final analytic sample included 32,602 delivery hospitalizations: 21,049 (64.6%) with opioid use disorder, 9472 (29.1%) with stimulant use disorder, and 2081 (6.4%) with co-occurring opioid and stimulant use disorder.

### 2.4. Definition of Variables

The primary outcome of interest was adverse pregnancy outcome (APO), structured as a composite measure to include: hypertensive disorders of pregnancy (HDPs), antepartum hemorrhage (AH), postpartum hemorrhage (PPH), preterm birth (PTB), and fetal growth restriction (FGR). HDPs included gestational hypertension, preeclampsia, eclampsia, and pre-existing hypertension with superimposed preeclampsia. AH included placenta previa, placental abruption, and other forms of antenatal hemorrhage as identified by ICD-10 codes.

Sociodemographic covariates included patient age, race and/or ethnicity, insurance payor type (governmental, private, other, and missing), and median household income for the patient’s ZIP code. Race and ethnicity in the HCUP-NIS are provided by HCUP Partner organizations as a single uniform variable. Given sample sizes, race and ethnicity were categorized as Black, White, Hispanic or Latino, other (including American Indian or Alaska Native, Asian or Asian American, and Native Hawaiian or Other Pacific Islander), and missing. Hospital characteristics, including hospital region, urban–rural location, bed size, and teaching status, were also considered [27].

### 2.5. Data Collection

All data were obtained from the publicly available HCUP-NIS database for the years 2016–2020. Diagnosis and procedure codes used to identify study exposures, outcomes, and covariates are detailed in Appendix A. The dataset was limited to records with complete data on all variables of interest.

### 2.6. Statistical Analysis

Survey-weighted proportions were computed for all variables of interest across the full sample and stratified by SUD exposure type (opioid use disorder, stimulant use disorder, and co-occurring opioid and stimulant use disorder). For categorical variables, survey-weighted frequencies and proportions are reported. For continuous variables, survey-weighted means with standard deviations are presented. Rao–Scott Chi-Square tests were used to compare the distribution of patient characteristics across exposure groups. Multivariable logistic regression analyses were performed to estimate adjusted odds ratios (aORs) and associated 95% confidence intervals (CIs) for the primary binary outcome of APOs (yes/no) across the entire cohort of pregnancies affected by opioid, stimulant, or co-occurring opioid and stimulant use disorder. All candidate covariates were entered simultaneously using a forced-entry approach. Covariates were selected a priori based on clinical relevance and the prior literature, and included patient age (categorical: <20, 20–29, 30–39, or ≥40 years), race and/or ethnicity (Black, White, Hispanic or Latino, other, or missing), insurance payor type (governmental, private, other, or missing), and median household income quartile for the patient’s ZIP code (0–25th, 26th–50th, 51st–75th, or 76th–100th percentile). White race, private insurance, and the 76th–100th income percentile served as reference categories. A two-sided significance threshold of α = 0.05 was applied for all hypothesis tests. Prior to multivariable modeling, model assumptions were assessed, and multicollinearity among covariates was evaluated using variance inflation factors (VIF); all VIF values were below the conventional threshold of 10, indicating no meaningful multicollinearity among the included predictors. Model fit was assessed using the Hosmer–Lemeshow goodness-of-fit statistic as implemented in PROC SURVEYLOGISTIC.

We additionally evaluated each SUD class as an independent predictor of APOs relative to pregnancies without SUD in the NIS cohort during the same timeframe. This model was adjusted for the same set of sociodemographic covariates described above, with no SUD serving as the reference category. All analyses accounted for the complex survey design of HCUP-NIS by incorporating hospital ID as a clustering variable, survey year, and NIS stratum as strata, and discharge weights as survey weights to produce national estimates [28]. Discharge weights were applied to all analyses to produce nationally representative estimates of the U.S. inpatient population. The PROC SURVEYFREQ and PROC SURVEYLOGISTIC procedures in SAS 9.4 (SAS Institute Inc., Cary, NC, USA). were used to implement survey-weighted analyses, incorporating the cluster, strata, and weight variables as specified by HCUP analytic guidelines. Records with missing data on any variable of interest were excluded from the analysis (complete case analysis); the proportion of records excluded on this basis is reported in the study flowchart (Figure 1). No adjustment for multiple comparisons was applied, given the exploratory and descriptive nature of the secondary outcome analyses; findings should be interpreted accordingly. Statistical analyses were completed using SAS 9.4 (SAS Institute Inc., Cary, NC, USA). The NIS database is de-identified, and hence, this study was deemed exempt by our IRB.

## 3. Results

From 2016 to 2020, a total of 32,602 delivery hospitalizations complicated by either stimulant or opioid use disorder or both (co-occurring opioid and stimulant use) were identified from the NIS database. Among these hospitalizations, the majority of patients were 20 to 30 years of age, identifying as White, and receiving types of government insurance. Delivery hospitalizations complicated by co-occurring stimulant and opioid use disorder increased each year from 2016 to 2019 (Table 1).

Of these admissions, 21,049 (64.6%) were complicated by opioid use disorder, 9472 (29.1%) by stimulant use disorder, and 2081 (6.4%) by co-occurring opioid and stimulant use disorder. Co-occurring opioid and stimulant use disorder was identified most often in hospitalizations of patients aged 20 to 30 years, identifying as White, receiving governmental insurance, and in the lowest income bracket. Co-occurring use was most often found in hospitalizations in the Western hospital region, at urban teaching centers, and at hospitals classified as large in size (Table 1).

**Figure 2:**
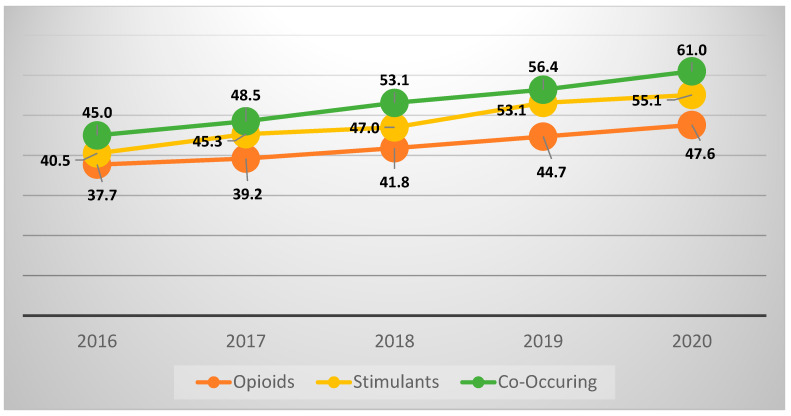
Adverse Pregnancy Outcome Rates (%) by Substance Use Disorder Type, National Inpatient Sample 2016–2020.

During the study period, there were a total of 14,713 (45.1%) pregnancy-related hospital admissions complicated by opioid, stimulant, or co-occurring use disorder with an associated diagnosis of an APO. The rate of APO was significantly highest among pregnancy delivery hospitalizations with co-occurring opioid use and stimulant use disorder (1136/2081—54.6%, *p* < 0.001), as compared with opioid use disorder (8923/21,049—42.4%) or stimulant use disorder alone (4654/9472—49.1%). The composite APO rate by substance use type and rate of individual APO sub-groups is demonstrated in Table 2. The rate of APO increased in subsequent years for all cohort groups. There was an increase of 16% from 2016 to 2020 in the rate of APO in pregnancy hospitalizations with co-occurring use disorder (Figure 2, Appendix B).

Among all patients with opioid, stimulant, or co-occurring opioid and stimulant use disorder, the major predictors of APO were an age greater than 40 (aOR 1.51; CI 95%, 1.48–1.53), Black race (aOR 1.56; CI 95%, 1.55–1.57), government insurance (aOR 1.01; CI 95%, 1.00–1.02) and lower income, with the largest income-related predicting factor being income in the 0–25th percentile (aOR 1.23; CI 95%, 1.22–1.24) (Table 3). After adjusting for relevant sociodemographic covariates, co-occurring opioid and stimulant use disorder was an independent predictor of APOs (aOR 3.65; CI 95%, 3.34–3.99) compared to hospitalizations without substance use. In comparison, opioid use disorder and stimulant use disorder were independent predictors of APOs with a less strong correlation, aOR 2.22 (CI 95%, 2.16–2.29) and aOR 2.89 (CI 95%, 2.77–3.02), respectively (Table 4).

## 4. Discussion

In this nationally representative study examining the impact of co-occurring use disorder on adverse pregnancy outcomes, we found co-occurring opioid and stimulant use disorders to present the highest risk for composite APOs compared to patients with opioid or stimulant use disorder alone. The prevalence of co-occurring opioid and stimulant use disorder doubled during the study period. Meanwhile, the rate of APO within hospitalizations with co-occurring use also increased by over 15%. Co-occurring use disorder had an over 13% and 5% higher rate of APOs compared to opioid use or stimulant use alone, respectively.

Despite the high prevalence of substance use in reproductive-aged women, there is limited data to help understand the impact of co-occurring use in pregnancy, especially in the context of deriving insights from a nationwide inpatient database. Co-occurring use disorder outside of pregnancy has been shown to have higher rates of overdose, with the highest rate seen with opioid plus cocaine use [29]. To our knowledge, among pregnancy data, a single study by Smid et al. has been performed, looking at co-occurring opioid and methamphetamine use disorder with severe maternal morbidity and mortality (SMM) in Utah. It demonstrated that pregnant patients with co-occurring use had an increased rate of SMM compared to individual use alone [25]. While this study was limited to a dataset from a single state and focused solely on SMM, our study utilizes a national dataset and focuses on a broad range of pragmatic APOs to critically examine the differential impact of single versus co-occurring SUDs. Our findings add to the evidence base in this area by providing additional insight into the perinatal risks involved, including structured guidance on the magnitude of impact. This provides crucial information that can help guide patient counseling and facilitate informed decision-making. Our findings are consistent with prior evidence, both in and outside of pregnancy, showing increased risks to persons with polysubstance use, with particular emphasis on the risks with stimulant plus opioid use.

Polysubstance use in pregnancy is on the rise in the United States [21,30]. Jarlenski and colleagues published on the trend of polysubstance use among pregnant patients with opioid use disorder. They found that polysubstance use among pregnant individuals with opioid use disorder increased from 60.5% to 64.1% from 2007 to 2016. More specifically, an increase in the prevalence of co-occurring opioid and amphetamine use among pregnant patients was seen by 255.4% in rural counties and 150.7% in urban counties [21]. An additional study by Jarlenski and colleagues published on the trends of all co-occurring use disorders in pregnancy hospitalizations from 2007 to 2016. They found that some of the highest prevalence of any co-occurring use disorder was seen in patients who used amphetamines or opioids, at 63% and 62%, respectively, although it should be noted that the most common additional substances used were tobacco and cannabis [30]. Describing these impressive rates of co-occurring substance use in pregnancy was the first step to isolating a problem, whereas our study adds another essential layer of data to this evidence base by providing actionable information about the magnitude of perinatal risk seen in pregnancies impacted by co-occurring opioid and stimulant use.

The impact of co-occurring SUD in pregnancy has been evaluated in a handful of other studies. Polysubstance use is suspected to cause a synergistic effect when two or more drugs are used together, with unclear impact on the pregnancy and neonatal outcomes [24]. Logue and colleagues examined APO rates in patients with opioid and cannabis use disorders in pregnancy from 2000 to 2018. In patients with cannabis use disorder, they found increased adjusted risks for PTB and AH. In patients with opioid use disorder, they found an increased risk of non-transfusion SMM, PTB, and AH. However, they did not separate these categories by patients with specifically co-occurring use [31]. Additional studies have evaluated co-occurring substance use in local populations. Tevis and colleagues showed that when comparing pregnant individuals with methamphetamine use, opioid use, or co-occurring opioid and methamphetamine use at a single treatment facility in Kentucky, individuals with co-occurring use were significantly more likely to have recent intravenous drug use and overdose prior to starting treatment [32]. Lastly, Smid and colleagues describe neonatal outcomes among pregnant individuals with opioid and methamphetamine use disorders and mental health diagnoses in Utah from 2014 to 2016. They found higher rates of neonatal abstinence syndrome in infants from mothers with co-occurring opioid use disorder and methamphetamine use disorder [33]. Despite previous research examining the relationship between co-occurring SUDs and pregnancy outcomes, specific data on the impact on adverse perinatal outcomes has been lacking. Therefore, a significant strength of this work is the ability to specifically address this critical gap in the evidence base.

This study has both strengths and limitations, most of which are inherent to utilizing a pre-existing national database. Utilization of the HCUP-NIS database allowed for evaluation of greater than thirty thousand pregnancies impacted by opioid or stimulant use, offering a perspective from a population health level. While this sample size allowed us to grasp a national representation of substance use in pregnancy in the United States, we are limited by the anonymity of the database and the inability to conduct further chart review. We were unable to confirm or refute inaccuracies in ICD-10 coding at the hospital or individual patient level, and recognize that coding may be susceptible to errors. We also acknowledge that most investigations evaluating the impact of perinatal SUDs would be remiss without a discussion of neonatal outcomes, including neonatal abstinence syndrome. However, the focus of this paper was APOs, particularly in the maternal context. The NIS database does not provide insights into neonatal outcomes and does not allow the linking of maternal data to neonatal information from related administrative datasets.

Our research has identified and described the increased risks in pregnancies affected by co-occurring stimulant and opioid use disorder. While our work focused on identifying perinatal risks in this population, we recognize that more work is needed to understand how best to predict individualized risk profiles in the context of perinatal SUD, using a risk stratification approach.

## 5. Conclusions

Our research has shown that pregnancies complicated by co-occurring stimulant and opioid use disorder are at a substantially greater risk for APOs when accounting for sociodemographic factors. Our analysis provides evidence to demonstrate that co-occurring SUD is an independent risk factor for APOs, which presents a higher risk profile than either individual substance alone. Our findings call for the need for increased awareness among clinicians and improved recognition of the heightened risks that patients with polysubstance use may face.

## Figures and Tables

**Table 1:** Patient demographics by drug type exposure.

Variable	All (*n* = 32,602)	Opioids (*n* = 21,049)	Stimulants (*n* = 9472)	Co-Occurring Use (*n* = 2081)
	*n* (%)	*n* (%)	*n* (%)	*n* (%)
Age (y)				
<20	534 (1.6)	223 (1.1)	294 (3.1)	17 (0.8)
20–<30	17,238 (52.9)	11,011 (52.3)	5081 (53.6)	1146 (55.1)
30–<40	14,047 (43.1)	9326 (44.3)	3846 (40.6)	875 (42.0)
40+	783 (2.4)	489 (2.3)	251 (2.7)	43 (2.1)
Race				
White	23,881 (75.8)	16654 (81.5)	5745 (63.3)	1482 (74.4)
Black	2288 (7.3)	1449 (7.1)	747 (8.2)	92 (4.6)
Hispanic or Latino	3404 (10.8)	1429 (7.0)	1707 (18.8)	268 (13.5)
Other	1931 (6.1)	903 (4.4)	879 (9.7)	149 (7.5)
Missing	1098	614	394	90
Insurance Payor				
Governmental	27,113 (83.3)	17,325 (82.4)	8022 (84.9)	1766 (85.1)
Private	3928 (12.1)	2940 (14.0)	800 (8.5)	188 (9.0)
Other	1514 (4.6)	764 (3.6)	628 (6.6)	122 (5.9)
Missing	47	20	22	5
Income				
0–25th percentile	12,388 (39.0)	7678 (37.0)	3935 (43.4)	775 (38.9)
26th–50th percentile	9516 (29.9)	6189 (29.8)	2757 (30.4)	570 (28.6)
51st–75th percentile	6648 (20.9)	4493 (21.7)	1719 (19.0)	436 (21.9)
76th–100th percentile	3253 (10.2)	2390 (11.5)	653 (7.2)	210 (10.6)
Missing	797	299	408	90
Hospital Region				
Northeast	5356 (16.4)	5074 (24.1)	204 (2.2)	78 (3.8)
Midwest	6809 (20.9)	4502 (21.4)	1942 (20.5)	365 (17.5)
South	11,691 (35.9)	8163 (38.8)	2907 (30.7)	621 (29.8)
West	8746 (26.8)	3310 (15.7)	4419 (46.6)	1017 (48.9)
Hospital Location and Teaching Status				
Rural	4989 (15.3)	2886 (13.7)	1831 (19.3)	272 (13.1)
Urban nonteaching	5419 (16.6)	3281 (15.6)	1813 (19.2)	325 (15.6)
Urban teaching	22,194 (68.1)	14,882 (70.7)	5828 (61.5)	1484 (71.3)
Hospital Bed Size				
Small	5908 (18.1)	3777 (17.9)	1822 (19.3)	309 (14.9)
Medium	8843 (27.1)	5979 (28.4)	2381 (25.1)	483 (23.2)
Large	17,851 (54.8)	11,293 (53.7)	5269 (55.6)	1289 (61.9)
Year				
2016	5749 (17.6)	4044 (19.2)	1456 (15.4)	249 (11.9)
2017	5898 (18.1)	3977 (18.9)	1620 (17.1)	301 (14.5)
2018	6129 (18.8)	3896 (18.5)	1841 (19.4)	392 (18.8)
2019	7467 (22.9)	4609 (21.9)	2324 (24.5)	534 (25.7)
2020	7359 (22.6)	4523 (21.5)	2231 (23.6)	605 (29.1)

All comparisons *p*-value < 0.001.

**Table 2:** Adverse pregnancy outcomes by substance use disorder exposure group.

Outcome	Opioid Use Disorder (*n* = 21,049)	Stimulant Use Disorder (*n* = 9472)	Co-Occurring Opioid & Stimulant SUD (*n* = 2081)	Total (*n* = 32,602)	*p*-Value
Composite APO, *n* (%)	8923 (42.4)	4654 (49.1)	1136 (54.6)	14,713 (45.1)	<0.001
Hypertensive disorders of pregnancy	3357 (15.9)	2294 (24.2)	494 (23.7)	6145 (18.8)	<0.001
Antepartum hemorrhage	879 (4.2)	525 (5.5)	150 (7.2)	1554 (4.8)	<0.001
Postpartum hemorrhage	802 (3.8)	486 (5.1)	120 (5.8)	1408 (4.3)	<0.001
Preterm birth	4493 (21.3)	2799 (29.6)	673 (32.3)	7965 (24.4)	<0.001
Fetal growth restriction	2069 (9.8)	434 (4.6)	131 (6.3)	2634 (8.1)	<0.001

APO = adverse pregnancy outcome; SUD = substance use disorder. All frequencies and percentages are survey-weighted. *p*-values from Rao–Scott Chi-Square tests comparing the distribution of each outcome across the three SUD exposure groups.

**Table 3:** Predictors of adverse pregnancy outcome among patients with opioid/stimulant/co-occurring opioid and stimulant use disorders.

	APO aOR 95% CI (LL, UL) *
Age (Reference = <20 years)	
20–<30	0.85 (0.84, 0.86)
30–<40	0.97 (0.96, 0.99)
40+	1.51 (1.48, 1.53)
Race (Reference = White)	
Black	1.56 (1.55, 1.57)
Hispanic or Latino	0.96 (0.95, 0.96)
Other	0.95 (0.94, 0.95)
Insurance Payor (Reference = Private)	
Governmental	1.01 (1.00, 1.02)
Other	0.89 (0.89, 0.91)
Income (Reference = 76th–100th Percentile)	
Income 0–25th Percentile	1.23 (1.22, 1.24)
Income 26th–50th Percentile	1.15 (1.14, 1.16)
Income 51st–75th Percentile	1.12 (1.11, 1.13)

aOR, adjusted odds ratio; adjusted for age, race, insurance, and income level; * All comparisons *p*-value < 0.001.

**Table 4:** Substance use subtype and risk for APOs.

	APO aOR 95% CI (LL, UL) *
Drug exposure (reference = no SUD)	
Any other type of SUD (excluding opioids or stimulants)	1.46 (1.46, 1.47)
Opioid use disorder	2.22 (2.16, 2.29)
Stimulant use disorder	2.89 (2.77, 3.02)
Co-occurring opioid and stimulant use disorder	3.65 (3.34, 3.99)

aOR, adjusted odds ratio; SUD, substance use disorder. adjusted for age, race, insurance, and income level; * All comparisons *p*-value < 0.001.

## Data Availability

Data were obtained from the Healthcare Cost and Utilization Project National Inpatient Sample, which is publicly available data.

## Abbreviations

The following abbreviations are used in this manuscript:

SUD	Substance Use Disorder
APO	Adverse Pregnancy Outcomes
SMM	Severe Maternal Morbidity
HCUP	Healthcare Cost and Utilization Project
NIS	National Inpatient Sample
HDPs	Hypertensive Disorders of Pregnancy
AH	Antepartum Hemorrhage
PPH	Postpartum Hemorrhage
PTB	Preterm Birth
FGR	Fetal Growth Restriction

## Appendix A

**Table A1:** Diagnosis and procedure codes used in the analysis.

Condition	ICD-10 Code
Pregnancy, Childbirth, and Delivery	O00.0, O10X, O11, O12X, O13, O14X, O15X, O16, O20X, O21X, O22X, O23X, O24X, O25, O26X, O28X, O29X, O30X, O31X, O32X, O33X, O34X, O35X, O36X, O40, O41X, O42X, O43X, O44X, O45X, O46X, O47X, O48, O60X, O61X, O62X, O63X, O64X, O65X, O66X, O67X, O68X, O69X, O70X, O71X, O72X, O73X, O74X, O75X, O80X, O81X, O82X, O83X, O84X, O85, O86X, O87X, O88X, O89X, O90X, O94, O95, O96X, O97X, O98X, O99X
Opioid Use	F1110, F11120, F11121, F11122, F11129, F1115, F11150, F11151, F11159, F11181, F11188, F1119, F1120, F1121, F11220, F11221, F11222, F11229, F1123, F1124, F11250, F11251, F11259, F11282, F11282, F11288, F1129, F1190, F11920, F11921, F11922, F11929, F1193, F1194, F11950, F11951, F11959, F11981, R781F11982, F11988, F1199
Cocaine Use	F1410, F14120, F14121, F14122, F13129, F1414, F1450, F14151, F14159, F14180, F14181, F14182, F14188, F1419, F1420, F1421, F14220, F14221, F14222, F14229, F1423, F1424, F14250, F14251, F14259, F14280, F14281, F14282, F14288, F1429, F1490, F14921, F14921, F14922, F14929, F1494, F14950, F14951, F14959, F14980, F14981, F14982, F14988, F1499, R782
Other Stimulant Use	F1510, F15120, F15121, F15122, F15129, F1514, F15150, F15151, F15159, F15180, F15181, F15182, F15188, F1519, F1520, F1521, F15220, F15221, F15222, F15229, F1523, F1524, F15250, f15251, F15259, F1523, F1524, F15250, F15251, F15251, F15259, F15280, F15281, F15282, F15288, F1529, F1590, F15920, F15921, F15922, F15929, F1593, F1594, F15950, F15951, F15959, F15980, F15981, F15982, F15988, F1599
Hypertensive Disorders of Pregnancy	O10X, O11X, O13X, O14X, O15X, O16X
Antepartum Hemorrhage	O20X, O44.0X, O44.1X, O44.3X, O44.4X, O44.5X, O45X, O46X, O67X
Postpartum Hemorrhage	O72X
Preterm Birth	O601X, O4201X, Z3A1X, Z3A2X, Z3A30-6
Fetal Growth Restriction	O365X, Z364, P050X, P051X, P052, P059

## Appendix B

**Table A2:** Adverse pregnancy outcomes by substance use disorder type.

	Opioids	Stimulants	Co-Occurring	*p*-Value
	N (%)	N (%)	N (%)	
**Year**				
2016–2020	8923/21,049 (42.4)	4654/9472 (49.1)	1136/2081 (54.6)	<0.0001
2016	1526/4044 (37.7)	590/1456 (40.5)	112/249 (45.0)	0.0202
2017	1558/3977 (39.2)	734/1620 (45.3)	146/301 (48.5)	<0.0001
2018	1628/3896 (41.8)	866/1841 (47.0)	208/392 (53.1)	<0.0001
2019	2060/4609 (44.7)	1234/2324 (53.1)	301/534 (56.4)	<0.0001
2020	2151/4523 (47.6)	1230/2231 (55.1)	369/605 (61.0)	<0.0001
